# Decision tree modeling predicts effects of inhibiting contractility signaling on cell motility

**DOI:** 10.1186/1752-0509-1-9

**Published:** 2007-01-29

**Authors:** Sourabh Kharait, Sampsa Hautaniemi, Shan Wu, Akihiro Iwabu, Douglas A Lauffenburger, Alan Wells

**Affiliations:** 1 Department of Pathology, University of Pittsburgh, Pittsburgh, PA 15213, USA; 2 Biological Engineering Division, Massachusetts Institute of Technology, Cambridge, MA 02139, USA; 3 Computational Systems Biology Laboratory, Institute of Biomedicine and Genome-Scale Biology Research Program, Biomedicum Helsinki, 00014 University of Helsinki, Finland

## Abstract

**Background:**

Computational models of cell signaling networks typically are aimed at capturing dynamics of molecular components to derive quantitative insights from prior experimental data, and to make predictions concerning altered dynamics under different conditions. However, signaling network models have rarely been used to predict how cell phenotypic behaviors result from the integrated operation of these networks. We recently developed a decision tree model for how EGF-induced fibroblast cell motility across two-dimensional fibronectin-coated surfaces depends on the integrated activation status of five key signaling nodes, including a proximal regulator of transcellular contractile force generation, MLC (myosin light chain) [Hautaniemi *et al*, Bioinformatics 21: 2027 {2005}], but we have not previously attempted predictions of new experimental effects from this model.

**Results:**

In this new work, we construct an improved decision tree model for the combined influence of EGF and fibronectin on fibroblast cell migration based on a wider spectrum of experimental protein signaling and cell motility measurements, and directly test a significant and non-intuitive *a priori *prediction for the outcome of a targeted molecular intervention into the signaling network: that partially reducing activation of MLC would increase cell motility on moderately adhesive surfaces. This prediction was indeed confirmed experimentally: partial inhibition of the activating MLC kinase (MLCK) upstream using the pharmacologic agent ML-7 resulted in increased motility of NR6 fibroblasts. We further extended this exciting finding by showing that partial reduction of MLC activation similarly enhanced the transmigration of the human breast carcinoma cell line MDA-213 through a Matrigel barrier.

**Conclusion:**

These findings specifically highlight a central regulatory role for transcellular contractility in governing cell motility, while at the same time demonstrating the value of a decision tree approach to a systems "signal-response" model in discerning non-intuitive behavior arising from integrated operation a cell signaling network.

## Background

Phenotypic cell behaviors are strongly governed by various extracellular cues, such as binding of cell surface receptors to soluble (*e.g*., growth factor, cytokine) and insoluble (*e.g*., extracellular matrix) ligands. Cue combinations can generate distinct cell behavioral responses by selectively inducing signal transduction pathway activation. It is rare, however, that a particular signal actuates only a single pathway; rather, the rule is for the activation of multiple divergent pathways that together hold potential to elicit numerous, often mutually exclusive, cellular responses. The cell phenotypic outcome may be considered to derive from a governing pattern of activation across the network comprising particular pathways in interconnected fashion. While the simplest hope might be to understand and manipulate cell behavior in terms of targeting an intervention at some "most important" molecular "switch", in reality there is a greater likelihood that such an intervention will impact the network as a whole much more broadly – and quite possibly with unexpected phenotypic outcome effects. Thus, systems biology approaches are now being conceived for application to signaling network control of complex cell responses, in order to gain rationale capability for predicting the effects of targeted interventions [1,2].

One vital cell functional behavior is that of motility induced by growth factors. This plays a key physiological role during organogenesis and wound healing. Further, induced cell motility is dysregulated in cancers leading to cancer progression and metastasis [3]. Thus targeting motility can be employed in the hope of limiting tumor dissemination [4]. But such targeting is a challenge as a ramifying network of signaling pathways lead to motility. While total inhibition of individual pathways leading to motility and subsequent invasiveness can be attained *in vitro*, this cannot be readily applied to the *in vivo *milieu, as inhibitor levels fluctuate due to pharmacodynamics. Furthermore, as these signaling networks are interrelated, alterations in one will lead to changes in many others. Thus, a clear delineation of the interplay of key proteins mediating cellular properties is crucial to future efforts aimed at drug discovery and individualized treatment [5,6].

Targeting growth factor-induced cell motility that drives tumor invasion is a challenge given its complexity. One approach towards understanding motility is to break it down into discrete and individual biophysical components [7,8]. The principal processes that are well studied include acquisition of cell directionality with a front and a rear end with lamellipodal protrusion (with PLCγ as the key signaling nexus) at the front, transcellular contractility (with PKCδ and MLC as molecular switches), and detachment of cell membrane at the rear (with m-calpain being the main regulator) [4]. Hence, productive migration ensues due to the repetitive cycling of these complex biophysical events in a temporally organized manner. It is evident that such a complex event is exhibited by a coordinated signal propagation and amplification/attenuation within existing intracellular proteomic networks. Our goal is to define how these key signaling switches governing cell migration can be targeted for modifying this cellular behavior, all the while recognizing the quantitative adaptations of the other pathways that will compensate for the subtotal interventions of a single pathway.

Computational modeling can compile and classify data sets in a quantitative manner and consequently provide testable predictions to extract vital information not readily apparent by conventional analytical techniques. In addition, mathematical models can expand data sets to proportions that can be used to make non-intuitive predictions related to biological responses [9]. We have previously offered [1] a decision tree modeling approach for understanding cell migratory events based on measurements of activation status of key intracellular signaling proteins. This study was inspired by earlier observations from Maheshwari *et al *[10] that elucidated the biophysical components of fibroblast migration across a range of different extracellular cues. Individual cellular biophysical processes including cell speed were measured across 8 different experimental conditions (4 different surface fibronectin (FN) concentrations and presence or absence of EGF). The observations indicated that cells move fastest upon EGF stimulation when the surface fibronectin concentration (or cell-substratum adhesiveness) is in the intermediate range whereas minimal motility was observed at the two extreme conditions. However, substratum fibronectin concentration (and also the extracellular matrix) alters motility not just by altering surface adhesiveness but also by actively signaling through the integrins towards downstream intracellular cascades [11]. Here, we have applied the methodology suggested in [1] to new signaling protein data sets at 1 h and 16 h in addition to 5 min, in order to construct a more powerful decision tree model capable of *a priori *prediction. The computational analysis suggested that the activation status of the contractility-related molecular switch, myosin light chain (MLC), as key to migration speed. Further, our results predicted that maximal speed would be achieved at intermediate levels of MLC activity. We test this prediction explicitly by modulating MLC activation status directly, finding successful experimental confirmation of a non-intuitive effect that an inhibitory pharmacological agent enhances migration speed – not only for the original model-foundation fibroblasts but also for an additional test case, breast tumor cells.

## Results

### Signaling protein data set across different surface fibronectin concentration in the presence of EGF

We aimed to elucidate the relative contributions of different signaling proteins in mediating biophysical migratory processes of adherent cells across different extracellular conditions. As a model system, we chose a mouse fibroblast line for which biophysical data included cell speed, membrane protrusion activity, cell spread area, surface adhesion, and membrane retraction (previously measured by Maheshwari *et al *[10]). Our new study employed 10 nM of EGF whereas the earlier Maheshwari study used 25 nM, but both of these concentrations are saturating for the EGF receptor level on these cells so can be assumed to be similar in their cellular effects. In addition, EGF was added to the cells for periods of 5 minutes, 1 hour and 16 hours to capture the entire (temporal) activation spectrum of signaling proteins, during the phases of acute effects, the transition to productive motility, and during sustained motility [10,12].

**Figure 1:**
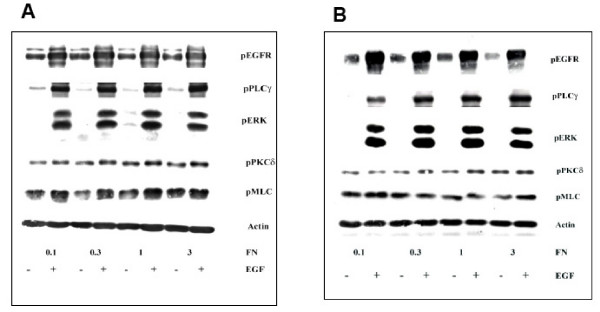
**Immunoblotting data for EGF treatment of 5 minutes (A) and 1 hour (B) across different fibronectin concentration of surfaces**. Tissue culture plates were coated with different fibronectin (FN) concentrations. NR6WT cells were grown on these surfaces for 24 hours in complete growth medium and quiesced for another 24 hours in medium containing 0.5% dialyzed FBS. EGF was added for a period of 1 hour, cells washed once with PBS and lysed. Cell lysates were resolved using SDS-PAGE and immunoblotted using specific antibodies for various phosphorylated proteins. At least 5 replicates for each signaling protein were created for polynomial modeling. Actin served as a loading control.

**Figure 2:**
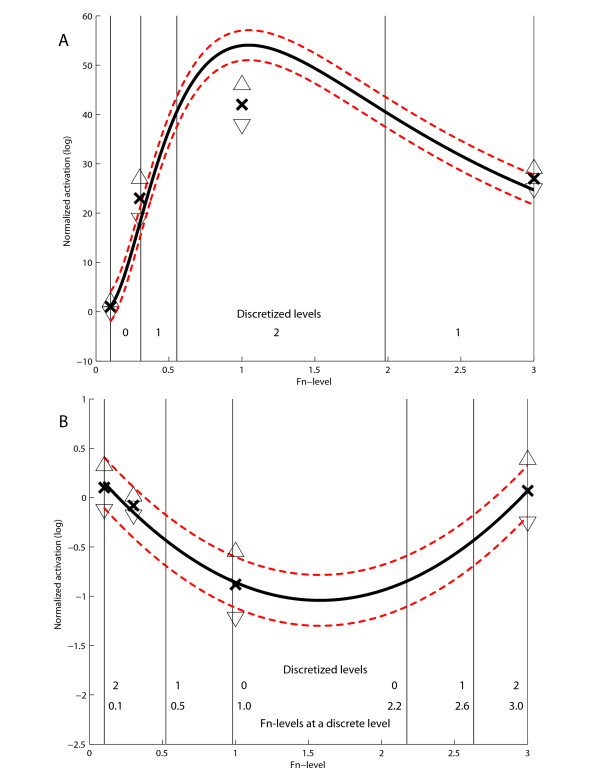
**Polynomial interpolation data for cell speed (A) and activated MLC (B) under EGF stimulation**. Crosses are actual measurements, upper and lower triangles are individual standard deviations, red lines denotes squared pooled standard deviation and the vast majority of the simulated cases fall between these lines. Cell speed is biphasic whereas activated MLC is inversely biphasic across fibronectin. Each variable is discretized into low, medium and high in accordance with the degree of polynomial.

Addition of EGF activated EGFR within minutes and this signal was transmitted downstream to signaling cascades measured (Figure 1A). Interestingly, the EGFR activation profile mirrored that of ERK within early time periods of EGF stimulation (5 minutes). ERK activation was robust immediately after addition of EGF compared to quiesced cells and remained significantly increased for over an hour (of EGF stimulus) with minimal change over different surface fibronectin concentrations (Figure 1B). Thus, ERK functioned like a 'switch' turned on dependent mainly on EGFR signaling. EGFR signaling also activated PLCγ and PKCδ, with their activation increasing linearly across increasing surface FN levels with resultant MLC activation downstream of PKCδ [13]. However, fibronectin does exert a significant influence on cell speed, as predicted [11], biphasic with surface adhesiveness (Figure 2A).

MLC activation begins within a few minutes of EGF stimulation and reaches a plateau at about 2 hours; increases were still appreciable up to 24 hours after EGF stimulus (unpublished observations). Interestingly, after 1 hour and longer exposure to EGF, MLC activity was inversely biphasic across fibronectin, with lowest levels at intermediate FN concentration (0.3 and 1 μg/ml) (Figures 1B and 2B). Thus, using these experimental conditions, we captured important quantitative and temporal trends of molecular activations.

### Decision tree model of signaling proteins predicts a critical role of myosin light chain (MLC) based cell contractility in mediating maximal cell migratory response

**Figure 3:**
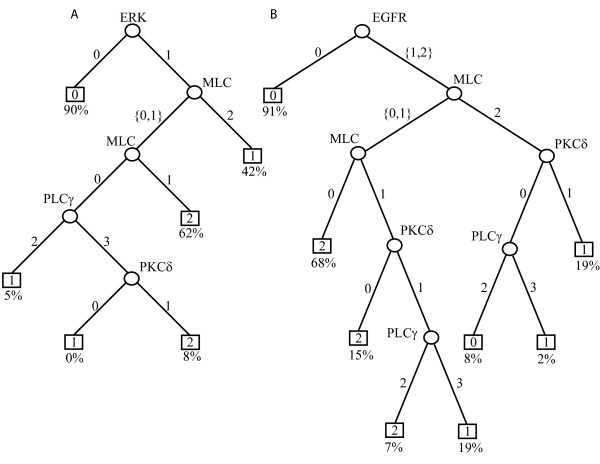
**Decision tree models from 5 minute (A) and 1 hour (B) EGF stimulation data set across fibronectin**. Round nodes denote (signaling proteins) whereas square nodes denote migration speed categories. Integers attached to the arc correspond to the split of the parent nodes. Under each migration speed category the fraction of cases explained by that classification rule is given. For example from (B), if EGFR is medium or high (1 or 2) and MLC is low (0), the migration speed category is 2 (high) and 68% of the observations (in the training set) for the high migration speed category can be explained by this rule.

A complex and well-orchestrated cellular response such as migration can only manifest from optimal quantitative activation/involvement of tens and hundreds of signaling proteins. Accordingly, it is important to address the relative contributions of such protein clusters in order to define the most significant switches that can be altered for therapeutic purposes. We constructed decision trees using the above five key signaling proteins activated on different levels of fibronectin by EGF that predicted the quantitative contribution of signaling proteins in dictating cell speed. The utility of decision trees is to identify prediction rules from the data and then illustrate them as a binary tree where each terminal node (leaf) corresponds to a class and other nodes represent measured variables. The decision trees obtained from three different EGF treatments (5 minute, 1 hour, and 16 hour measurements) yielded different classification efficiencies of observations from the training data set. The 5-minute decision tree accounted for approximately 70% of observations from the 1000 independent validation data sets correctly (Figure 3A), whereas the 1-hour decision tree had an explanatory power of greater than 75% (Figure 3B). The 16-hour decision tree could account for only less than 60% of the observations from the validation data sets, however, so was eliminated for further consideration; this result was comforting given the expectation that signaling network activity should be upstream of the longer-term cell behavior. Lastly, generating a decision tree of similar simplicity using data across the three time periods did not increase the predictive accuracy above that of the 1-hour tree (data not shown). For our purposes of testing capability for *a priori *prediction of effects of signal inhibition, we focused on the 1-hour model because of its superior performance with the independent validation data sets.

Contractile force production is enabled through the actin-myosin coupling upon activation of regulatory myosin light chains [13,14]. While each of the 'crucial molecules' that govern motility have been characterized, decision tree analysis is useful in predicting which of these molecules, and therefore which of the biophysical processes they controlled, were hierarchically important in governing motility. As such, since the 1-hour decision tree had the maximum classification accuracy, we utilized it to extract important predictions. Interestingly, after EGFR activation, MLC mediated contractility was the most crucial ingredient in mediating maximal motility. According to the predictions from the 1-hour decision tree (Figure 3B), the cells move with highest speed when following EGFR activation MLC phosphorylation is low; in training set 68% of the situations in which cells move with high speed can be explained with this rule alone. In other words, lowering MLC activation and resultant contractility to a subtotal level apparently leads to enhanced cell motility whereas total MLC inhibition can abrogate cell motility. While the effects of total MLC inhibition on cell motility have been intuitive and published by Iwabu *et al *[13], the biphasic dependence of cell migration (speed) upon subtotal inhibition of MLC is non-intuitive and novel. Moreover, it is an especially significant prediction for targeted therapeutics because it indicates that subtotal versus total abrogation of a key signaling pathway node can have drastically opposite cell responses.

### Subtotal inhibition of MLC activation increases cell speed

Our model predicted that subtotal lowering of MLC activation would increase fibroblast cell speed. Our experimental data set indicated that while cell speed showed a biphasic response, MLC activation was inversely biphasic across fibronectin concentration of the surfaces. Thus, at the two extreme conditions, where surface fibronectin was either too low (0.1 μg/ml) or too high (3 μg/ml), cell migration speed was minimal. From our polynomial model, these two conditions corroborated with surface FN concentrations below 0.522 μg/ml or greater than 2.6 μg/ml. At these two conditions there is apparent dysregulation in the balance between the substratum adhesion strength versus contractility; *i.e*., despite high MLC activation in both conditions, there is too little substratum adhesion at 0.1 μg/ml while it is in excess at 3 μg/ml [10]. Thus, at 0.1 μg/ml, contractility supersedes adhesion strength whereas this phenomenon is reversed at the condition of 3 μg/ml of surface fibronectin.

**Figure 4:**
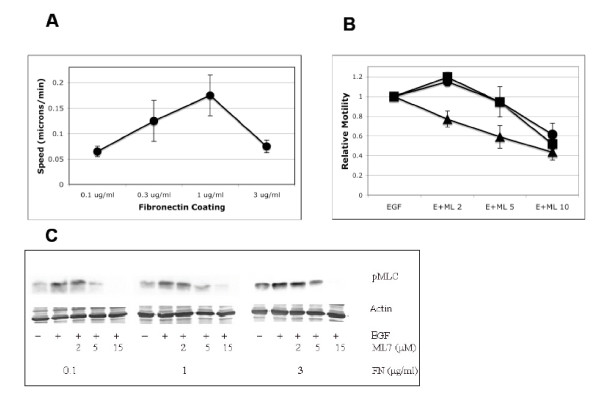
**Subtotal inhibition of myosin light chain kinase increase cell migration via single-cell tracking**. NR6WT fibroblasts were grown on fibronectin-coated surfaces coated and quiesced in serum-restricted conditions for 16 hours. After drug inhibition and/or EGF stimulation, single cells were tracked for up to 20 hours and their migration speeds analyzed using Visible, developed by Reify Corporation. Each experimental condition is the average ± SEM of 15–20 cells. (A) Four concentrations of fibronectin were used (0.1, 0.3, 1, 3 μg/ml) and the biphasic relationship between speed and fibronectin was indeed reproduced via our single-tracking setup and analysis. (B) Under higher fibronectin conditions (1 and 3 μg/ml), partially inhibitory ML-7 concentrations increases migration speed while further inhibition reduces the closure of the in vitro wound. At low fibronection concentrations (0.1 μg/ml) further reduction of MLC activation reduced wound closure. Shown are mean ± SEM of four experiments performed in triplicate and normalized within run to no ML-7 control speeds. In comparison to no ML-7 treatment, P < 0.05 for 2 μM ML-7 treatments on 1 and 3 μg/ml fibronectin; the decreases in speed were also statistically significant at higher ML-7 concentrations for all three surfaces. 0.1 μg/ml FN are triangles, 1 μg/ml FN are circles and 3 μg/ml FN are squares. (C) Attenuation of MLC activity using graded concentrations of MLCK inhibitor, ML-7. MLC activity is completely abrogated at concentrations greater than 15 μM. Three FN levels (low, medium and high concentrations) are shown for simplicity. Shown is one of three representative experiments.

To test the model predictions under such conditions, we employed a well-characterized MLCK inhibitor, ML-7, to measure fibroblast migration speed under the same extracellular conditions (4 FN concentrations -/+ EGF). Such a downstream inhibitor was chosen (over PKCδ inhibitor Rottlerin) because it is MLC kinase-specific and hence the resultant cellular responses can be attributed directly and specifically to MLC inhibition since PKCδ is involved in diverse cellular responses in addition to motility [15]. In addition, fibronectin ligandation can activate MLC-based contractility, likely independent of PKCδ. These considerations are likely reflected in the decision tree analysis wherein MLC lies hierarchically above PKCδ. We initially measured cell migration on fibronectin using the 'scratch assay' under a range of ML-7 concentrations within the culture medium containing saturating levels of EGF. In parallel, immunoblotting analysis of activated MLC (with EGF treatment) showed a linear decrease in phosphorylated MLC levels with increasing ML-7 concentration (Figure 4). Under the same conditions and as predicted by the decision tree model, lower ML-7 concentration (2–3 μM) increased fibroblast migration compared to EGF alone at fibronectin concentration of 1 and 3 μg/ml (Figure 4). Greater inhibition led to the predicted decrease in motility. We validated this fibroblast migration speed using single cell tracking under the same experimental conditions. Speed was measured as the distance traveled by an individual cell over a given period of time (10 hours) [10]. We found that a partially inhibitory ML-7 concentration in the presence of EGF increased cell migration distance as well as speed relative to EGF alone (from 0.076 ± 0.014 microns/min to 0.118 ± 0.018 microns/min, N = 14, P < 0.05, testing partial inhibition on 3 μg/ml fibronectin). This greater than 50% increase in individual cell speed accords with earlier studies that show that *in vitro *wound healing assays minimize increases in cell speed. The outcomes of these experiments determining the effect of partial reduction of MLC activation are in accordance with the predictions from our decision tree model.

### Subtotal inhibition of myosin light chain activity increases migration of cancer cells

**Figure 5:**
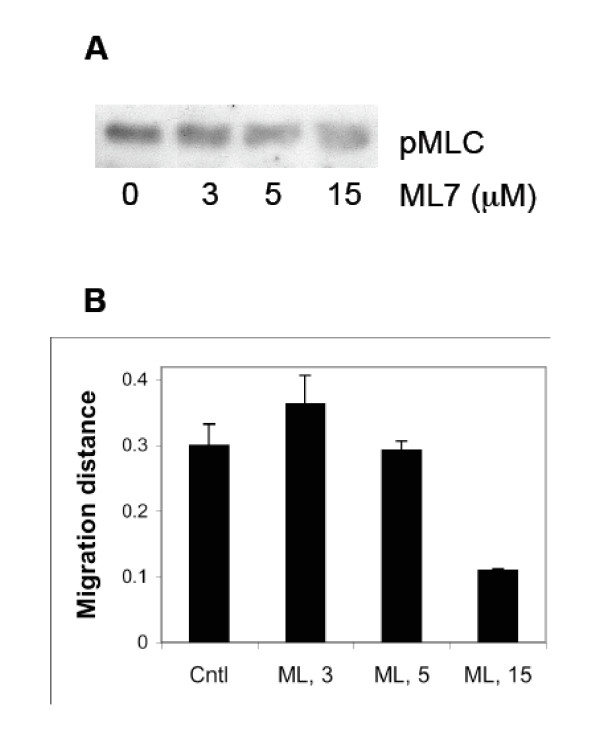
**Subtotal inhibition of myosin light chain activation increases migration of cancer cells**. (A) MDA-MB-231 breast cancer cells were grown in complete medium, quiesced for 24 hours in serum deprived medium (with 0.5% dialyzed FBS) and incubated with varying concentrations of MLCKinase inhibitor, ML-7. Cells were lysed and immunoblotting of lysates was carried out using SDS-PAGE to detect activated levels of MLC. Shown are one of three similar blots.(B) MDA-MB-231 cells were grown in complete medium until they formed a confluent monolayer. The medium was then replaced by 0.5% dialyzed FBS containing quiescent medium for 24 hours. The monolayer was scraped using a sterile pipet tip, washed three times with PBS and migration of cells in the denuded area was assessed over a period of 24 hours in the presence of increasing doses of MLCKinase inhibitor, ML-7. Shown are mean ± SEM of three experiments each performed in triplicate. In comparison to no ML-7 treatment, P < 0.05 for 3 and 15 μM ML-7 treatments.

To assess whether our predictions of hierarchical control could be extended to a different application of EGF-induced cell motility behavior, we utilized the MDA-MB-231 invasive human breast cancer cell lines and measured their migratory response across a range of MLC kinase (and hence MLC) inhibition. These cells overexpress EGF receptor and actively exhibit autocrine stimulatory loops that drive their migration and invasiveness [16]. In accordance with the findings in fibroblasts, migration of MDA-MB-231 cells was substantially higher when the medium contained low concentration (3 μM) of ML-7 as compared to diluent alone (Figure 5). The term 'low' or 'medium' in relation to ML-7 concentration is obtained from titrated inhibition of MLC under those concentrations and varies within different cell types; i.e. for NR6WT cells, 10 μM of ML-7 is high whereas the same is 'medium' for MDA-MB-231 cells. In other words, the amount of MLC downregulation that is achieved by 10 μM in NR6WT cells is approximately similar to that achieved by 20 μM in MDA-MB-231 cells. Migration was completely lowered when ML-7 concentration completely abrogated MLC activity (20 μM).

## Discussion and conclusion

The vast majority of diseases are now appreciated to be "complex"; *i.e*., they arise from alterations within multiple molecular regulatory pathways. Signaling pathways represent an especially critical domain for pathological dysregulation, as they contain forward- and reverse-feedback cascades that can act as signal amplifiers, transmitters, or distributors to a multitude of highly-connected protein nodes across numerous pathways within a network. Thus, multiple signaling proteins with interactive activity profiles govern phenotypic cell behavioral phenomena underlying normal physiology and pathology. Altering cell behaviors is difficult without a thorough understanding of how these signaling switches work in relation to each other. While enormous data sets are available for biological conditions, such data sets have not been integrated to provide information about the interlinked and branched signaling networks. Therefore, targeted therapies often fail because cells utilize parallel and alternative pathways to mediate the necessary biological functions. Identification and modulation of key signaling nexi from such complex networks can alter cell behaviors and yield favorable responses [17,18].

We utilized here decision tree analysis to identify the crucial effectors of cell motility depending upon a set of extracellular cues. Fibronectin was selected since NR6WT fibroblast express α5β1 integrin receptors that are actively involved in cell signaling during motility. Also, these being adhesion receptors provide a counter-balance against the motogenic EGF receptor that is overexpressed in these cell lines. Such adhesion versus motility balance is present *in vivo *environments, where motility of cells is dictated by the cellular ecology, cell-substratum and cell-cell adhesion profile, extracellular matrix components along with a spectrum of soluble and matrix-embedded extracellular stimuli [19,20]. Our model was based on the quantitative measurements of five signaling proteins that are activated downstream of the EGFR and are known to mediate key biophysical events of motility. Arguably, such a model could suffer from predictive power due to the possible exclusion of other key signaling proteins (such as FAK, calpain, *etc*). However, our model achieved 75% accuracy for independent validation data sets, which is more than twice expected by random association. Future experiments are aimed at incorporating other key signaling proteins within this foundational decision tree model.

Our decision tree model clearly identified MLC-mediated contractility as a key regulatory biophysical event during EGF induced motility. This does not mean, however, that disrupting other cellular events, such as PLCγ-based lamellipodal protrusion, will not abrogate motility. The utility of a decision tree model is to predict the switches that upon disruption can produce highly significant responses and illustrate them as a hierarchical logic. Decision trees represent non-linear depictions of contributory influencers and do not imply hierarchies or linkages between the constituent molecules or events. A decision tree model may also suggest molecules that need to be inhibited together to alter the cell phenotypic behavioral outcome. In our model that was based on 5-minute and 1-hour EGF stimulation data set (Figure 3), contribution by ERK was masked by similar activation profile observed with EGFR. This does not mean that ERK is not vital in motility since disrupting ERK reduces migration [21] but rather means that the contribution of ERK activation was captured by measuring EGFR activation and did not provide further information to the prediction in itself. Further, the model predicted is in accordance with Glading *et al *[22] that motility requires functional ERK activation since 90% of cells that migrated could be explained to operate using this rule alone (Figure 3). Furthermore, even the 5-minute data set resulted in a predictor with 70% accuracy, although maximum motility is observed at least 4 to 8 hours after EGF addition [10,12]. This may derive from the fact that 5-minute measurements can capture activation trends of important molecules such as ERK that are indispensable for cell migration but are usually attenuated at 1 to 2 hours after EGF stimulus when motility has started becoming a stable biophysical response. Such transient activation is sufficient to elicit motility since ERK transmits the signal downstream towards the final effectors of motility before attenuation. Additionally, the model indicates that ERK functions like an 'on-off' switch during motility: if ERK (and/or the EGFR) is active, the cells will move depending upon the profile of other signaling proteins but if ERK is inactive, the motility is practically negligible since 90% of cells with minimal motility could be predicted by this rule alone (Figure 3). This also points to a new proposition: targeting MLC and ERK together to retard cell migration.

Our model, non-intuitively predicted that lowering MLC activation, but not totally abrogating it, can paradoxically increase cell speed. These predictions held true in the population based 'scratch assay' that assessed cell migration distance as well as single cell tracking that assessed migration speed, under different concentrations of MLCKinase inhibitor, ML-7. An especially important consequence is that of subtotal inhibition of MLCKinase under higher fibronectin concentration of substratum increased cell speed (Figure 4) whereas under lower substratum adhesive conditions (0.1 μg/ml), further reduced it (data not shown). Motility is a function of optimum balance between cell-substratum adhesion versus cell contractility that enables cells to break some cell-substratum adhesions but form newer ones as the cell moves [23]. This is evident at intermediate fibronectin concentration of surfaces in our experiments [10]. The adhesion-contractility balance is impaired at the two extreme conditions where too little adhesion precludes a cell from generating sufficient adhesions for locomotion; hence further lowering of contractility even by subtotal inhibition of MLC further reduces motility. On the other hand, too much surface adhesiveness (fibronectin of 3 μg/ml) maintains a cell in an unproductive situation due its inability to detach. This is because higher surface fibronectin promotes excessive integrin receptor engagement evenly on the surface rather than keeping it selective at focal adhesions. Cell-substratum adhesiveness is governed by a combination of ligand concentration, receptor number or ligand-receptor affinity, with maximum motility (and cell speed) occurring at intermediate level of cell-substratum adhesion strength [11,23]. Thus, a higher FN concentration results in a cell stuck to the surface with a high intrinsic contractile force. In such situations, any decrease in contractility can be predicted to increase cell motility by reinstating the adhesion versus contractility balance and enabling cell detachment, breakage of focal adhesions with formation of new ones. This was indeed confirmed by our initial experiments using 'scratch assay' and observed in single cell tracking experiments.

These findings have profound implications for therapy. Identifying key nodes enables quantitative manipulations using pharmacologic methods for specifically desired cellular responses. It also points to the importance of how these signaling proteins are regulated stoichiometrically. Our predictions held true even when applied to breast cancer cells, where subtotal inhibitory doses of ML-7 promoted cell migration. While a complete abrogation of MLC can be beneficial in limiting tumor cell motility and hence invasion, partial inhibition using lower pharmacological doses can paradoxically increase tumor cell motility and invasion leading to devastating consequences. This further points to the importance of applying newer modeling approaches to fully characterize the role of signaling cascades in mediating cellular behaviors. Such understanding will enable precise therapeutic targeting of key signaling nodes and open the door to individualized 'patient-tailored therapy' [17].

## Methods

### Cell culture

NR6WT cells expressing human EGF receptor (EGFR) were maintained in modified Eagle's medium-α containing (MEMα) 7.5% fetal bovine serum (FBS) and 1% of each of the following: penicillin/streptomycin, L-Glutamine, non-essential amino acids and sodium pyruvate (all from GIBCO). The medium contained 350 μg/ml of G418 as a selection agent for human EGFR. Cells were quiesced in a medium containing 0.5% dialyzed FBS for 24 hours before addition of EGF. The MDA-MB-231 invasive human breast cancer cell line was maintained in RPMI 1640 medium (GIBCO) containing 10% FBS and 1% penicillin/streptomycin. Migration and immunoblotting assays were conducted by quiescing the cells in a medium containing 0.5% dialyzed FBS for 24 hours prior to experimentation.

### Reagents and antibodies

Antibodies used to detect activated status of EGFR (phosphorylated Tyr1173), ERK (phosphorylated Thr202/Tyr204), PKCδ (phosphorylated ser643), and myosin light chain (phosphorylated ser19) were obtained from Cell Signaling technology (Danvers, MA). Activated status of phospholipase Cγ (PLCγ) was probed using a rabbit polyclonal antibody against phosphorylated tyrosine 783 residue obtained from Santa Cruz Biotechnology (Santa Cruz, CA). ML-7 was utilized as MLCKinase specific inhibitor and was purchased from Calbiochem, EMD Biosciences (La Jolla, CA).

### Preparation of fibronectin-coated surfaces

Fibronectin coating concentrations of the surfaces were 0.1, 0.3, 1 and 3 μg/ml. Tissue culture plates were incubated with fibronectin at required concentrations diluted in PBS at room temperature for a period of 2 hours. The plates were washed once with PBS and incubated with 1% bovine serum albumin for another 1 hour to block non-specific protein binding during the course of the experiment. The plates were washed three times with PBS and cells plated directly in complete growth medium over these surfaces.

### Quantitative immunoblotting for signaling protein data

NR6WT mouse fibroblasts engineered to express human EGFR were utilized for our baseline modeling studies. These cells are derived from the 3T3 lineage, are devoid of an endogenous EGF receptor and serve as an excellent model system to study EGFR mediated cell migratory events. Equal number of NR6 WT cells were plated on fibronectin coated surfaces and allowed to grow in MEMα containing 7.5% fetal bovine serum (FBS) for 24 hours, by which time cells reached about 90% confluence. Subsequently, cells were quiesced in media containing 0.5% dialyzed FBS for another 24 hours, to minimize the effect of exogenous growth factors present in the serum. Cells were either lysed in the quiescent medium without any exogenous human EGF or stimulated with 10 nM (saturating concentration) of human EGF for either five minutes, one hour or 16 hours. Such time frames were selected to capture the entire spectrum of signaling protein activation during the motility response [12]. After stimulation, cells were washed once with ice cold PBS, and then lysed in lysis buffer containing 50 mM HEPES, pH 7.4, 150 mM NaCl, 1% Triton X-100, 1 mM Na Vanadate and 10% glycerol supplemented with protease inhibitors including 1 μg/ml Leupeptin, 1 μg/ml Aprotinin and 1 mM phenylmethylsulfonylfluoride (PMSF). Cell lysates were quantified using Biorad protein assay. Equal amount of total proteins were mixed with the loading buffer containing 4% SDS (w/v), 0.1 M Tris-HCl, pH 6.8, 20% glycerol, 0.2% Bromophenol blue and 5% β-mercaptoethanol, boiled for 5 minutes and then loaded on either 7.5% (for analysis of pPKCδ, pERK, pEGFR, pPLCγ) or 15% (for pMLC) SDS polyacrylamide gels. Cell lysates were resolved by electrophoresis and subsequently transferred onto nitrocellulose membranes, after which, membranes were immunoblotted with specific antibodies to detect the specific proteins or their activated phospho-protein forms. Immunoblots were quantified with the NIH image analysis densitometry software. The software generates an area plot for each protein band, the density of which represents the amount of the protein in each lane. In the signaling protein experiments, the quantitative values generated represented the activated status of a protein since the proteins detected were in their activated or phosphorylated state. At least 5 replicates were analyzed for each protein at each timepoint; all immunoblots performed were analyzed to capture the full extent of the noise inherent in such measurements [1].

### Data preprocessing

Prior to polynomial modeling and decision tree analysis, the data were thoroughly preprocessed by normalization and quality-control approaches described in [1]. First, densities in each band were divided by the value of the first lane (*Fn *= 0.1 and *EGF *= 0) for each immunoblot. After this between-band normalization, the numbers within an immunoblot become comparable to other immunoblots since the experimental conditions in each of the experiment were kept constant. For quality control purposes, the bands were also within-band normalized: all protein conditions in a band without exogenous EGF were divided by the value with *EGF *= 0 and *Fn *= 0.1, while all protein conditions in a band with exogenous EGF were divided by the value with *EGF *= 1 and *Fn *= 0.1. The within-band normalization ensures that proteins under the same EGF condition within a band are comparable. Prior to normalization all basal values below 250 were converted to 250 in order to prevent division by a small value that is likely due to noise. After normalization, all the values were log_2_-transformed.

Normalization was followed by the ANOVA based quality control approach and statistical outliers were discarded [1]. Each variable (signaling protein) had at least five replicate values (except PKCδ for 16 h that had four replicates) after quality control for polynomial modeling.

### Development of computational model

Our goal was to create a predictive model that is able to predict migration speed as a function of signaling proteins, and provide insight on what signaling proteins could key elements governing migration. Accordingly, we chose the decision tree methodology since decision trees both show the predictive structure of the signaling proteins and are fairly accurate classifiers [24]. As there are eight observations across EGF and fibronectin concentrations per variable, a classifier based on these data only would be weak. Thus, we first used polynomial modeling to find parametric models for the variables to capture protein activity as a function of fibronectin. These models were then used to simulate data in an interpolative manner across fibronectin concentrations and used in the classification.

### Polynomial interpolation of signaling protein data set

Prediction algorithms in general require large training and validation data sets to ensure that the resulting predictor is reliable and the results reproducible. Therefore, we developed mathematical models that capture signaling protein activity and migration speed profiles as a function of fibronectin concentrations. Variables (signaling proteins and migration speed) were modeled using the polynomial function family. Polynomial functions family was chosen because it allows for modeling of a large spectrum of different trends. To choose degree for a polynomial model, we applied normalized maximum-likelihood (NML) approach, which is an implementation of the minimum description length (MDL) principle and aims at describing the data best without overfitting [25]. Technical details of the NML approach in estimating polynomial degrees are derived and discussed in [25].

The polynomial models were constructed separately for the values with or without exogenous EGF. As the first value (no exogenous EGF and fibronectin concentration of 0.1 μg/ml) in each immunoblot was used in normalization, the polynomial modeling for data without exogenous EGF was done with three data points, whereas data with exogenous EGF was modeled with four values. Accordingly, the maximum polynomial degree in the NML modeling step was set to two. The resulting polynomial estimates (β) and squared pooled standard errors (*s*^pooled^) used in the simulations are given in Additional File 1.

We used the resulting polynomial models to create 10000 simulated training sets (58002 cases in each data set) and 1000 validation data sets (5802 cases in each data set). Data for each signaling protein and migration speed were then discretized using the Lloyds algorithm [26], which minimizes the average quantization noise power and is essentially the same as the *k*-means clustering method. Thus the only parameter needed in the Lloyds discretization method is the number of discrete categories. In this study the number of discrete categories was chosen to be the number of the polynomial estimates for 5 min data set. For example, EGFR for 5 min has three parameters, so EGFR is discretized to low (0), medium (1) and high (2) phosphorylation levels. We have illustrated the discrete regions for cell migration speed and MLC in Figure 2.

### Decision tree construction

Decision tree predictors aim to uncover the predictive structure of a classification or prediction problem while still maintaining good prediction accuracy. Here, we used the classification and regression trees (CART) approach [24]. A more detailed description of the use of the CART in modeling migration speed using signaling proteins is given in [1].

The CART results in a decision tree where interior nodes represent signaling proteins and leaves migration speed classes. Each interior node is actually a question that splits the data into two subsets. For example, the first question in the 1 h decision tree (see Figure 3) is whether activity of EGFR is low (0). Accordingly, all cases where EGFR is low go to left (29005 cases), while the rest (28995 cases) go right. The rule "*EGFR is low*" results in 20790 cases having slow migration speed of 22883 cases belonging to slow migration category (91%). Further, as in the data set split to the right there are only 8211 cases belonging to medium speed and 4 to fast speed classes, the data are not split further and the rule "*EGFR is low*" predicts slow migration speed. If EGFR is medium or high, however, the set of 22883 cases is split further until sufficiently good prediction accuracy is achieved. The parameters for the decision tree learning were as follows. Purity function was the Gini-index, variables having more than five cases were considered for a split and prior probability for *i*th class was obtained by dividing the number of the cases of *i*th class by the total amount of observations. The cost of a misclassification from high to low speed was 2, medium to high or low was 1, and the cost for correct classification was 0. After constructing a decision tree, we applied the cost-complexity pruning method [24] to avoid over-fitting. All computations were performed using MATLAB v6.5 with Statistics toolbox.

We simulated 10000 training data sets and used them to learn decision tree predictors. These 10000 decision tree predictors were then applied to 1000 independent validation data sets and the predictor giving the best classification accuracy was chosen. For 5 min, 1 h and 16 h data sets, the best decision tree predictors achieved 70%, 75% and 57% accuracy, respectively.

### In vitro migration assay

Cell migration was measured as the distance traveled by the cells into a cellular area. Cells were seeded in 6-well tissue culture plates for a period of 24 hours in growth medium. Cells were quiesced for another 24 hours in serum free medium at which time cells formed a confluent monolayer. A denuded area was created by scraping with a pipet tip, washed three times with phosphate buffered saline (PBS) to remove dead cells, and kept under serum free conditions throughout the experiment. EGF at 10 nM (and inhibitors or diluent as indicated) was added to the serum free medium. Cells were then photographed using an inverted microscope immediately following scraping (0-hour condition) and 24 hours later (24-hour condition) in exactly same three different areas. The photographs were merged and analyzed using Adobe Photoshop program to determine the average distance traveled by the cells in 24 hours. All experiments were performed in triplicate.

### Single cell tracking for cell speed analysis

For final validation of cell migration, individual cell speeds were measured using time-lapse videomicroscopy. 6,000 cells were plated on each fibronectin-coated DeltaT imaging dish (Bioptechs) in 2 ml of assay medium containing 0.5% dialyzed FBS and 1% BSA. 16 hours post-seeding, the medium was replaced with 3.2 ml of fresh assay medium. In migration versus fibronectin validation studies, the replacement medium contained 10 nM EGF. In MLC inhibition studies, the replacement medium contained 0, 2, 4, or 10 μM ML-7 (MLCK inhibitor), and 10 nM EGF was added 45 minutes after ML-7 exposure. The plates were then sealed with a vacuum grease-lined coverglass lid and placed in a heated stage insert for a Ludl 99S008 motorized stage on a Zeiss Axiovert 35 microscope. Three fields of cells, with five to ten cells per field, were tracked by recording an image for each field every 15 minutes for up to 20 hours. Individual cell speeds were calculated using Visible (Reify Corporation, Cambridge, MA), which determines speeds by generating instantaneous velocity vectors for each pixel of the image that is associated with a cell. We found that cell speeds reach a steady-state 4–6 hours after adding EGF as previously reported [10], and as such the reported speed ± SEM for each condition is an average of 15–20 cells' speeds at each time point between 6 to 8 hours.

## Authors' contributions

SK performed many of the final signal activation measurements and the tumor cell motility experiments; he also wrote the initial manuscript drafts. SH constructed the decision trees as well as quality controlled the data; he contributed to the initial ideations and writing. SW performed the single cell tracking experiments and contributed experimental design and writing of the manuscript. AI performed the initial signal activation measurements in the fibroblasts, and shaped the origins of the project. DAL and AW provided overall guidance, initial conception of the project, integrated the varied aspects, and finalized the communications. All authors reviewed the data and contributed interpretations. All authors agree to the submitted manuscript.

## Supplementary Material

Additional file 1 Polynomial estimates and pooled error calculations for model/data fits. Each of the experimental data sets (5 minutes, 1 hour, and 16 hours) involves interpolating polynomials; the polynomials corresponding to each set, and the associated error in model data-fitting, are listed here. Click here for file
