# Supplementary material for: Decision tree modeling predicts effects of inhibiting contractility signaling on cell motility
Source: BMC Syst Biol. 2007 Jan 29;1:9. doi: 10.1186/1752-0509-1-9 (PMC1839898; doi:10.1186/1752-0509-1-9)
Supplement: Additional file 1 — Polynomial estimates and pooled error calculations for model/data fits. Each of the experimental data sets (5 minutes, 1 hour, and 16 hours) involves interpolating polynomials; the polynomials corresponding to each set, and the associated error in model data-fitting, are listed here. [file 1752-0509-1-9-S1.doc]

Tables 1-3. Polynomial estimates and standard deviation determinations for signaling protein measurements

Supplementary Table 1: Polynomial estimates and pooled error for 5min data set.

|  | β (EGF = 0) | *s*^pooled^ (EGF = 0) | β (EGF = 1) | *s*^pooled^ (EGF = 1) |
| --- | --- | --- | --- | --- |
| EGFR | 0.24x – 0.02 | 0.10 | 1.9 | 0.15 |
| ERK | 0.51 | 0.25 | 4.1 | 0.20 |
| MLC | 0.20 | 0.04 | 0.08x + 0.48 | 0.04 |
| PKCδ | 0.06 | 0.06 | 0.32 | 0.04 |
| PLCγ | 0.36x – 0.86 | 2.6 | 0.22x + 2.38 | 0.38 |

Supplementary Table 2: Polynomial estimates and pooled error for 1h data set.

|  | β (EGF = 0) | *s*^pooled^(EGF = 0) | β (EGF = 1) | *s*^pooled^ (EGF = 1) |
| --- | --- | --- | --- | --- |
| EGFR | -0.44 | 0.19 | 3.7 | 0.83 |
| ERK | -0.89 | 0.96 | 3.4 | 0.42 |
| MLC | -0.86 | 0.16 | 0.55x^2^ – 1.7x + 0.32 | 0.07 |
| PKCδ | -0.26 | 0.11 | 0.12x + 0.23 | 0.09 |
| PLCγ | 0.70x – 2.0 | 1.24 | 0.16x + 2.6 | 0.20 |

**Supplementary Table 3: Polynomial estimates and pool**ed error for 16h data set.

|  | β (EGF = 0) | *s*^pooled^(EGF = 0) | β (EGF = 1) | *s*^pooled^ (EGF = 1) |
| --- | --- | --- | --- | --- |
| EGFR | 0.11x + 0.29 | 0.14 | 1.8 | 0.07 |
| ERK | -0.54 | 0.29 | 1.2 | 0.36 |
| MLC | 0.47 | 0.17 | 1.2 | 0.11 |
| PKCδ | -0.36 | 0.05 | 0.4 | 0.02 |
| PLCγ | 0.42x – 0.01 | 0.36 | 2.5 | 0.49 |
